# Prevention and removal of membrane and separator biofouling in bioelectrochemical systems: a comprehensive review

**DOI:** 10.1016/j.isci.2022.104510

**Published:** 2022-06-02

**Authors:** Grzegorz Pasternak, Aleksander de Rosset, Natalia Tyszkiewicz, Bartosz Widera, John Greenman, Ioannis Ieropoulos

**Affiliations:** 1 Department of Process Engineering and Technology of Polymer and Carbon Materials, Wroclaw University of Science and Technology, 50-344 Wrocław, Poland; 2 Centre for Research in Biosciences, Department of Applied Sciences, University of the West of England, BS16 1QY Bristol, UK; 3 Water and Environmental Engineering Group, University of Southampton, SO17 1BJ Southampton, UK

**Keywords:** Surface chemistry, Biochemistry, Biochemical Engineering, Materials science engineering

## Abstract

Bioelectrochemical systems (BESs) have made significant progress in recent years in all aspects of their technology. BESs usually work with a membrane or a separator, which is one of their most critical components affecting performance. Quite often, biofilm from either the anolyte or catholyte forms on the membrane, which can negatively affect its performance. In critical cases, the long-term power performance observed for microbial fuel cells (MFCs) has dropped by over 90%. Surface modification and composite material approaches as well as chemical and physical cleaning techniques involving surfactants, acids, hydroxides, and ultrasounds have been successfully implemented to combat biofilm formation. Surface modifications produced up to 6–7 times higher power performance in the long-term, whereas regeneration strategies resulted in up to 100% recovery of original performance. Further studies include tools such as fluid dynamics-based design and plasma cleaning. The biofouling area is still underexplored in the field of bioelectrochemistry and requires systematic improvement. Therefore, this review summarizes the most recent knowledge with the aim of helping the research and engineering community select the best strategy and discuss further perspectives for combating the undesirable biofilm.

## Graphical abstract

**Figure:**
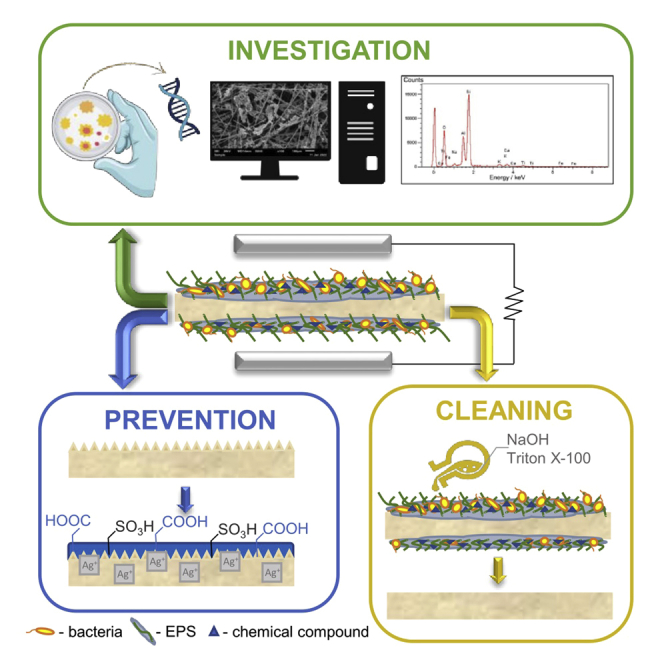

## Introduction

Bioelectrochemical systems (BESs) employ live microorganisms involved in various types of electrochemical reactions such as electricity production from wastewater or other types of waste in microbial fuel cells (MFCs) (Bennetto, 1990). In slightly modified reactors, bioelectrochemical synthesis of valuable compounds takes place, known as microbial electrosynthesis or electrofermentation (Rabaey and Rozendal, 2010; Sonawane et al., 2021; Roy et al., 2022). BESs have also been used for water desalination, electrolysis for the production of hydrogen or methane, and the recovery of metals (Cao et al., 2009; Sleutels et al., 2012; Kokko et al., 2017; Szydlowski et al., 2022).

**Figure 1:**
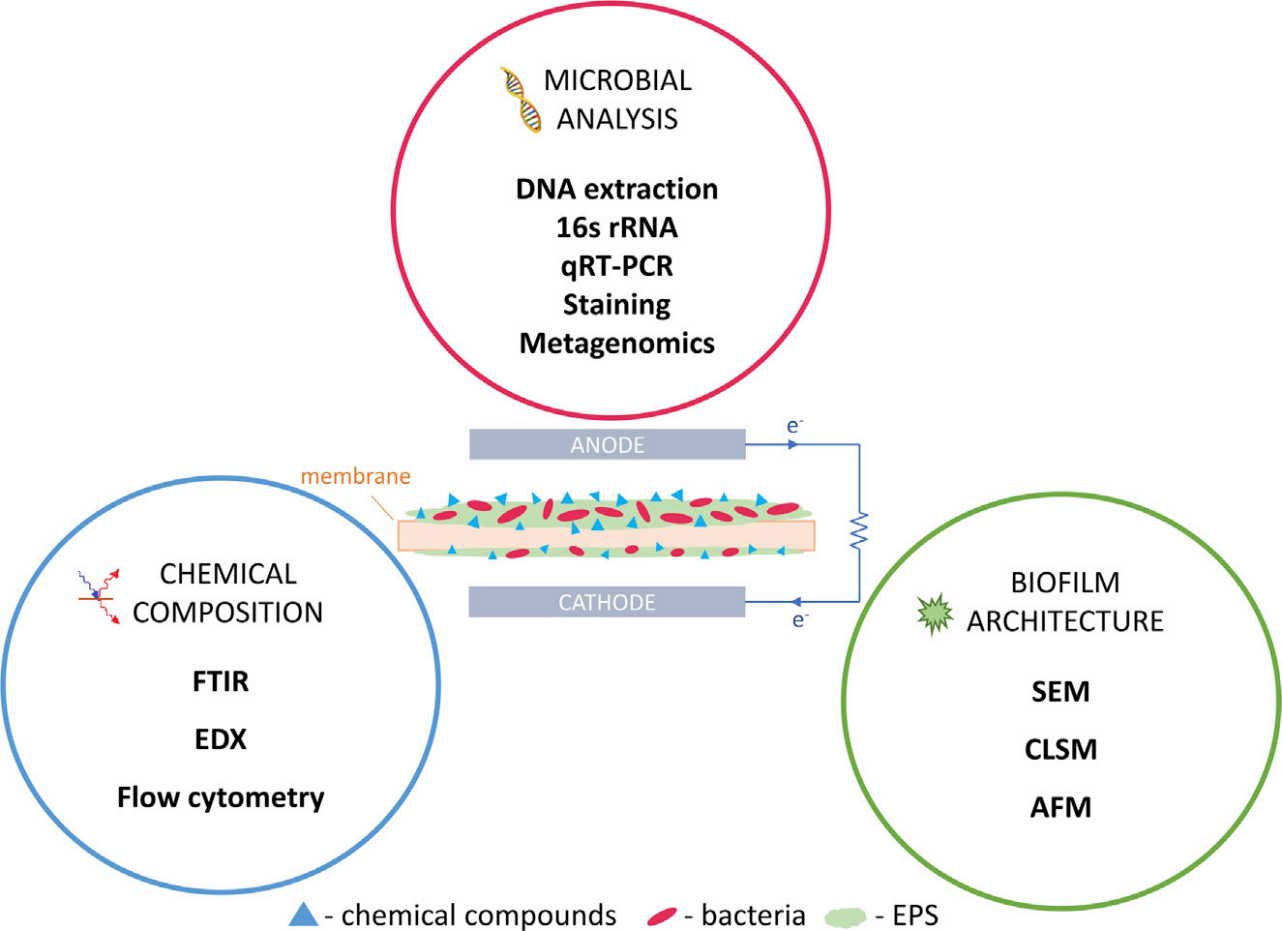
Summary of biofouling layer characterization methods

In all of the aforementioned systems, designed for specific applications, the vast majority of the designs are supplied with a membrane or separator. Their purpose is to create a physical barrier that prevents short-circuiting, oxygen and substrate cross-over between cathode and anode electrodes while maintaining the transfer of cations. These membranes are in direct contact with the organic and inorganic compounds, which inevitably leads to biofouling. Biofouling is a phenomenon (Figure 1) based on the aggregation of microorganisms, their metabolites, called extracellular polymeric substances (EPS), and inorganic salts (Dhar and Lee, 2013). Membranes, depending on the type of structural material, are divided into organic, inorganic, and mixed types. The first type is based on polymers, e.g., Nafion or sulfonated polymers. Several other types of polymers have also been investigated. Although the natural polymer-based membranes offer some unique features, they may be susceptible not only to biofouling but also deterioration (Pasternak et al., 2019). In contrast, synthetic polymers such as expanded polystyrene may offer long term durability but also longer start-up times (Mathuriya and Pant, 2019). The inorganic separators group is dominated by ceramic separators, whereas the third group comprises composite membranes (Dharmalingam et al., 2018; Pasternak et al., 2021). Ceramic separators offer good power performance but also high porosity, which may lead to high oxygen back-diffusion and substrate crossover, inducing the effects of biofouling (Pasternak et al., 2016a; 2016b). Interactions between chemical compounds that build membranes and the bacteria/EPS matrix result in the formation of a highly adherent coating (Xu et al., 2020). In contrast to biofilm formation on the anode, biofilm on the membranes is not beneficial for the performance of BESs. Such a biofilm layer makes the membrane less permeable for cations and contributes to the increased internal resistance of the system. The decrease in ion conductivity results in lower power density levels in MFCs (Dharmalingam et al., 2018) (Ji et al., 2011).

The biofouling layer is mainly composed of microorganisms. Therefore, to mitigate this phenomenon, it is crucial to inhibit the growth of bacteria and the production of their metabolites on the membrane surface. To achieve this objective, various strategies and modifications may be implemented in the BES-based process that could be based on antibacterial components, which kill microorganisms on the membrane surface, or antiadhesion molecules, which prevent the formation of bonds between microorganisms and components of the membrane (Leong et al., 2013). Other membrane physicochemical features that have an impact on bacterial adhesion include hydrophobicity, roughness, and the membrane surface charge (Pichardo-Romero et al., 2020). Although several examples have been published in the field of biofouling prevention in membrane science and technology, only limited knowledge is available for bioelectrochemical systems, and some of these aspects were discussed in a recent review (Koók et al., 2019). Herein, we summarize the most current knowledge in this underexplored field, focusing on biofouling monitoring, assessment, prevention, and removal methods, and discuss possible future tools that may help to combat this phenomenon.

## Biological insights

Characterization of the properties of the membrane and fouling layer requires the selection of appropriate research methods. In this section, various types of approaches and their applications will be discussed. The summary of biofouling investigation methods is provided in Figure 1, and a comparison of these methods with observed results in bioelectrochemical systems is shown in Table 1.

**Table 1:** Biofouling investigation techniques and observed effects

MFC type	Membrane type	Investigation technique	Observed effects	Reference
SCMFC	Nafion-117	• IEC and conductivity measurements, • FTIR-ATR, • SEM, EDX, • CLSM imaging, • Polarization tests	• FTIR analysis shows amide groups, which indicates the occurrence of proteins and suggests the involvement of biological fouling. • Microorganisms in the fouling layer are dominated by rod-type species. • Membrane fouling increases the internal resistance of the MFC and decreases the power output.	(Xu et al., 2012)
DCMFC	Nafion	• AFM imaging • SEM • EDX	• Morphology of the bacteria growing on the membrane was more diverse than that on the anode surface.	(Choi et al., 2011)
SCMFC	Nafion-117	• BCA assay • DNA extraction • Bacterial 16s rRNA • Archaeal 16s rRNA • Internal resistance measurements	• No biofilm was observed on the cathodic side of the membrane. • Methanogenic and archaeal species were detected on the anodic side. • The amount of biomass on the anodic side: 246 μgBSA/cm2.	(Kircheva et al., 2015)
	RhinoHide		• The biofouling layer was observed on both sides of the membrane. • Denitrifying the population of bacteria was detected on the cathodic side. • The amount of biomass on the anodic side: 309 μgBSA/cm2. • Increased permeability for microorganisms.	
OsMFC	TFC-FO	• SEM • EDX • CLSM • cytometry	• SEM images show different morphologies of the fouled membrane compared to the modified membrane. • Thickness of the biofouling layer: 69.87 ± 2.67 μm.	(Lu et al., 2020)
	TFC-FO modified with AgNP		• Thickness of the biofouling layer: 58.19 ± 1.24 μm. • The amount of polysaccharides, proteins, and microbes was less compared to that on the unmodified membrane.	
DCMFC	Nafion-115	• DNA extraction • 16s rRNA analysis • Ionic conductivity	• The ionic conductivity of the PSEBS SU22 membrane was higher compared to Nafion. • The observed range of OTUs in both membranes indicates relatively low richness values. • Aerobic species were detected on the membranes.	(Koók et al., 2021)
	PSEBS SU22			
SCMFC	SPEEK + TiO_2_	• Hemocytometer • SEM • EIS	• The higher hydrophilicity of the membrane reduced the adhesion of microbes on the membrane surface. • Microorganisms attached to the membrane were dominated by rod-type species and had a uniform morphology. • Higher proton conductivity was observed for the modified membrane in comparison to that observed for SPEEK and Nafion.	(Narayanaswamy Venkatesan and Dharmalingam, 2015)
SCMFC	Nafion™/SBA15-SO_3_H10	• SEM • Polarization test • EIS • Internal resistance	• SEM images show isolated and sparse rod-shaped microorganisms in the fouling layer. • The membrane surface was covered with a thinner biofouling layer compared to Nafion™ and Nafion™-SBA-15. • The biofouling extent of the membranes is in agreement with the power results.	(Angioni et al., 2016)
DCMFC	PSEBS DABCO AEM	• EIS • IEC	• The ionic conductivity of the fouled modified PSEBS DABCO AEM membrane was higher when compared to fouled Nafion PEM.	(Koók et al., 2020)

SCMFC – single chamber MFC, DCMFC – dual chamber MFC, OsMFC – osmotic MFC.

### Microbial communities' identification

To study the biodiversity of the membrane-colonizing microorganisms, some methods of molecular biology or analytical chemistry may be used. The composition of the microbial community depends on the type of inoculum, type of biocatalyst, *i*.*e*., mixed or pure culture, BES operating conditions, and the nutritional regime (Saratale et al., 2017). Knowledge of the local microenvironment could be important in finding out how to prevent the formation of biofilm. In addition, such knowledge might also be helpful during the development of new membrane modification techniques.

Molecular characterization of the microbial community could be based on the extraction of DNA and sequencing of its appropriate fragments (Takada et al., 2018). A commonly used method for bacteria comprises sequence analysis of genes encoding 16s rDNA, which contain variable regions. Diversity among the 16s rDNA regions in different species of bacteria even provides a strain-specific pattern of sequences. More details on this methodology are given by Kamel et al. (Kamel et al., 2020) and Xu et al. (2020). Bioinformatic analysis provides data about the phylogenetic connections between microorganisms and finally leads to identification of bacterial strains. This method may be a useful tool for the recognition of bacteria directly responsible for the biofouling of membranes. In a recent study by Xu et al. (2020), it was demonstrated that the integration of BES with ultrafiltration (UF) membrane, results in a complete change in the microbial composition present on the membrane. The content of the genera responsible for EPS production of *Zoogloea* and *Methyloversatilis* was significantly reduced in the BES integrated system. This is also confirmed by the measurement of EPS content by the CLSM method, where it can be observed that the number of proteins and polysaccharides in the integrated BES system was significantly less than in the control system. In addition, it was shown that the microbial community contained significantly more electroactive bacteria such as *Rhodocyclaceae*, *Oxalobacteraceae*, *Comamonadaceae*, and *Rhodospirillaceae*. Their growth was promoted by the introduction of the bioelectrochemical system. Kircheva et al. (2015) investigated the bacterial community on two types of membranes in a single chamber MFC after 8 months of operation. The similarity between bacterial communities obtained from Nafion-117 and RhinoHide membranes was only 15.4%. At the species level, the communities were significantly different, suggesting that the membrane type in BES has a large impact on the variety of microorganisms attached to the membrane surface.

To carry out quantitative DNA analysis, qPCR has been established as the standard tool. qPCR stands for quantitative Real-Time – Polymerase Chain Reaction. Amplification and quantification of DNA samples enable the identification of specific microorganisms occurring even at low numbers on the membrane surface (Zhang and Fang, 2006),(Dattatraya et al., 2017). However, because of the presence of free extracellular DNA also from dead cells, the number of bacteria could be overestimated, which was confirmed by Kim et al. (Kim et al. (2021).

To examine bacterial growth on the membrane, cellular staining may be applied. The most widely used dye is DAPI (4′6-diamidino-2-phenylindole). Staining allows one to observe cell nucleic acids under the microscope (Chae et al., 2008). In the previously cited work, DAPI staining was used and the treated DNA was visualized using the CLSM method. The control system was found to contain 74% more DNA material on the membrane compared to the membrane of the hybrid BES-UF integrated system (Xu et al., 2020). Structures within microbial cells may also be stained with FITC (Fluorescein isothiocyanate) and ConA (Concanavalin A). As a consequence, with FITC, proteins and amino-sugars from cells and EPS could be visualized, whereas ConA stains glycoproteins. Combining images in which the test material is stained with the above-mentioned dyes gives a holistic view of the biofouling layer on the membrane, which allows one to identify the individual elements of the layer (Xu et al., 2020). To distinguish live and dead cells on the surface, other specialized dyes must be used. For instance, Kim et al. (Kim et al. (2021) used SYTO9 and propidium iodide for the evaluation of bacterial viability, whereas Pasternak et al. (Pasternak et al., 2016a; 2016b) used acridine orange instead of SYTO9. The biofouling layer of the membrane-air-cathode assembly was composed of 80% inactive cells.

Another method used to assess the microbial diversity in the biofouling layer is metagenomics assessment. This approach combines molecular biology methods with a bioinformatics toolbox. This procedure, specifically for the MFC system, was recently described by Kook et al. on a sulfomethylated membrane based on polystyrene (Koók et al., 2021), where the principal component analysis (PCA) was performed on the relative abundances of the main bacterial orders. To investigate microbial diversity, the Shannon (*H′*) and Simpson (λ) diversity indices are also calculated. PCA analysis of the samples obtained from MFCs operated with two different membranes (Nafion 115 and sulfomethylated cation exchange membrane PSEBS SU22) and with two different types of inoculum (municipal wastewater collected in spring and autumn) indicated that the bacterial composition of the biofouling layer depends more on the type of inoculum than the type of membrane used. In contrast, Kircheva et al. (Kircheva et al. (2015) found a strong impact of the type of membrane on the microbial diversity. In their work, two types of membranes were used along with synthetic consortia supplemented with activated sludge. The PCA and Shannon analysis revealed significant differences in the biodiversity of the Nafion-117 and microporous polyethylene (RhinoHide®) biofouling layers. Furthermore, in the aforementioned work of Kook et al., most of the bacteria identified in the biofouling layer were aerobic. The growth of aerobic microorganisms on the membrane surface causes a reduction of oxygen concentration in the anode chamber. This may actually improve the performance of the electroactive bacteria (EAB) on the anode surface because power production is inhibited by oxygen.

### Biofilm architecture investigation

Microscopic techniques are commonly used to visualize the structure of the biofilm formed on the surface of the membrane. The most popular technique used to study membrane surface morphology is scanning electron microscopy (SEM) [16], [36], [37], [38]. Through the use of SEM techniques, it is possible to assess the degree of development of bacterial biofilm and measure its thickness. Miskan et al., 2016a) proved that the longer the operating time of the MFC, the more complex the architecture of the biofilm formed on the membrane. Two months after the start of the experiment, only single bacterial cells, mostly rod-shaped, were observed on the surface of the membrane; however, after 4 months of operation, the biofouling layer was significantly more complex and its thickness was around 165 μm, whereas after 6 months, its thickness was as much as 250 μm. Chae et al. (Chae et al. (2008) observed that biofilm formed on the membrane surface facing the anode exhibits a more diversified structure compared to that observed on the anode electrode. The reason for this is probably the oxygen permeability of the Nafion membrane, which promotes the growth of both aerobic and anaerobic bacteria. Venkatesan and Dharmalingam (Narayanaswamy Venkatesan and Dharmalingam, 2015) investigated the effect of membrane modification with nanoparticles on the membrane properties. Incorporation of TiO_2_ nanoparticles increased the hydrophilicity of the membrane, resulting in fewer bacterial cells adhering to the membrane and forming a biofouling layer, as confirmed by SEM images. They also visualized the biofouling layer formation process in MFCs, starting from free bacterial cells to the complex structure composed mainly of rod-shaped bacterial cells and extracellular polymer substances. Another insight into biofilm architecture is given when one couples the SEM technique with EDS/EDX mapping, as discussed in the subsequent section.

A second approach for visualizing bacteria attached to the membrane is based on CLSM (confocal laser scanning microscopy). By utilizing various dyes, components of the biofouling layer could be distinguished and observed by CLSM. Through the use of CLSM, it is possible to obtain a 3D image of the components included in the membrane biofilm. Furthermore, this method provides an easy way to visualize both dead and live cells on the membrane (Lu et al., 2020). Thus, the quantity and distribution of bacteria, EPS, and other metabolites can be determined (Zhu et al., 2016),(Xu et al., 2012). In their investigation, Xu et al. (2020) proved that the use of the CLSM technique allowed a comparison of protein and polysaccharide contents between membranes. Furthermore, the thickness of the biofouling layer could also be measured by CLSM [40]. As indicated in the study by Lu et al. (Lu et al. (2020), modification of the membrane with silver nanoparticles reduced the thickness of the biofouling layer from around 70 to 58 μm.

A third, less commonly used method for visualization of the membrane biofilm structure is by means of AFM (Atomic Force Microscopy). The image obtained by this method may sometimes lead to misleading observations. As shown in the study by Choi et al. (Choi et al. (2011), the biofouling layer on the membrane in the AFM analysis was relatively thinner than the images obtained by SEM. However, the diversity of components in the layer was noticeable and it could be concluded that the bacteria on the membrane were more morphologically diverse than those on the anode surface [41]. However, this method might be time-consuming, and only a relatively low area is investigated because of this time limitation. The great advantage of using AFM is its resolution, which may be indispensable when investigating the structural elements of microorganisms.

### Chemical composition of the biofouling layer

Investigating the chemical composition of the biofouling layer requires the use of various analytical techniques and provides important information about the properties of its superficial layer. The techniques most commonly used for this purpose are spectroscopic methods, one of the more popular of which is Fourier transform infrared spectroscopy (FTIR) (Xu et al., 2012, 2020; Ghasemi et al., 2013; Zhang, Hui and Han, 2015). The FTIR method uses infrared radiation to scan samples and identify functional groups, and finally chemical compounds. Analysis of the obtained spectrum delivers a molecular fingerprint of the tested sample. A very common approach is to compare the composition of the membranes before and after use. This allows the researcher to determine what substances have accumulated during the operation of the bioelectrochemical system. The components most frequently identified in the biofouling layer are proteins, fatty acid components, phospholipids, and polysaccharides. Based on chemical types, the presence of bacteria and extracellular polymeric substances can be detected on the membrane surface (Miskan et al., 2016b; Pasternak et al., 2019).

To perform quantitative chemical characterization of fouled membrane samples, the EDX method might be used. Energy-dispersive X-ray spectroscopy allows for the determination of which elements are in the sample and an estimation of their concentration. Multiple EDX analyses of contaminated membranes from BES have indicated the presence of precipitated inorganic salts (Zhu et al., 2016), (Xu et al., 2012),(You et al., 2019). EDX is frequently coupled with SEM analysis (Choi et al., 2011; Pasternak et al., 2016a, 2016b). The composition of inorganic salts on the membrane depends strictly on the type of inoculum and the composition of the feeding medium. In the work by Lu et al. (Lu et al. (2020), where wastewater was used as feed for MFC, a high diversity of elements was found in the biofouling layer, including C, O, Na, Mg, Al, P, S, Cl, K, Ca, Fe, and Cu. The combination of data obtained from both methods allowed the researchers to conclude that microorganisms in the biofouling layer are present in the surroundings of EPS, iron oxides, and inorganic salts (Zhang, Hui and Han, 2015). Such an approach was investigated by Sevda et al. (Sevda et al. (2013), who compared commercial Zirfon and Fumasep separators, both of which revealed signs of biofouling. Although a higher accumulation of carbon elements was observed for Zirfon during the experimental period, this membrane still outperformed Fumasep because of its overall lower resistance (3.8 Ω and 22.8 Ω, respectively).

Another analytical approach is flow cytometry, which is used to detect and define the morphological features of molecules or cells. This method could be used to determine the amount of live/dead bacteria attached to the fouled membrane (Malaeb et al., 2013) and was used for monitoring the regeneration process of conductive cathodes, painted directly over porous ceramic membranes (Pasternak et al., 2016a; 2016b).

### Indirect, electrochemical methods

The biofilm accumulation causes deteriorating changes in BESs electrochemical parameters, among which the resistance of membranes is the most prominent. This parameter can be directly measured using electrochemical impedance spectroscopy (EIS). However, it is worth remembering that this technique will provide information on the cumulative effect of chemical and biological fouling as well as other physical and chemical changes in the membrane structure, which may lead to changes in resistance. This technique has been applied in several studies such as the recent work of Pasternak et al. (Pasternak et al. (2021) where EIS was carried out at the end of a 3-month experimental period to assess the long-term performance of the MFCs. They found that the fouling and biofouling phenomena have a negative impact on the resistance. Membranes with the highest overall resistance values demonstrated the highest number of microorganisms on the cathodes, which was confirmed by the use of the colony-forming-unit (CFU) technique. The highest proportion of biofouling was found for the modified ceramic membranes: 373-ML and 468-CMP, which were, respectively, 1.8 ∗ 10^8^ and 3.5 ∗ 10^7^ CFU/cm^2^. They were accompanied by the highest total resistances above 800 and 1800 Ω, as compared to unmodified membranes. This is also confirmed by the work of Ghasemi et al. (Ghasemi et al. (2013), where an MFC with a biofouled untreated Nafion-117 membrane exhibited 2034 Ω of internal resistance. This was significantly higher when compared with a pretreated membrane – 824 Ω. The occurrence of biofouling can also be observed when the open circuit voltage (OCV) and power density are measured over time. These parameters are also reduced as a consequence of biofouling which is an immediate signal of biofouling occurring in MFCs (Xu et al., 2012).

## Surface modification approach

Long-term solutions to mitigate biofouling on MFC membranes include optimizing the operating conditions, changing hydrophilic-hydrophobic properties, and roughness of the membrane surface, doping biocides into the membrane matrix and thus creating antifouling coatings on the membrane surface (Noori et al., 2019). The surface properties of membranes can be influenced during their synthesis or by modifying the existing membranes.

### *In situ* strategies to improve the antibiofouling properties of membranes

**Figure 2:**
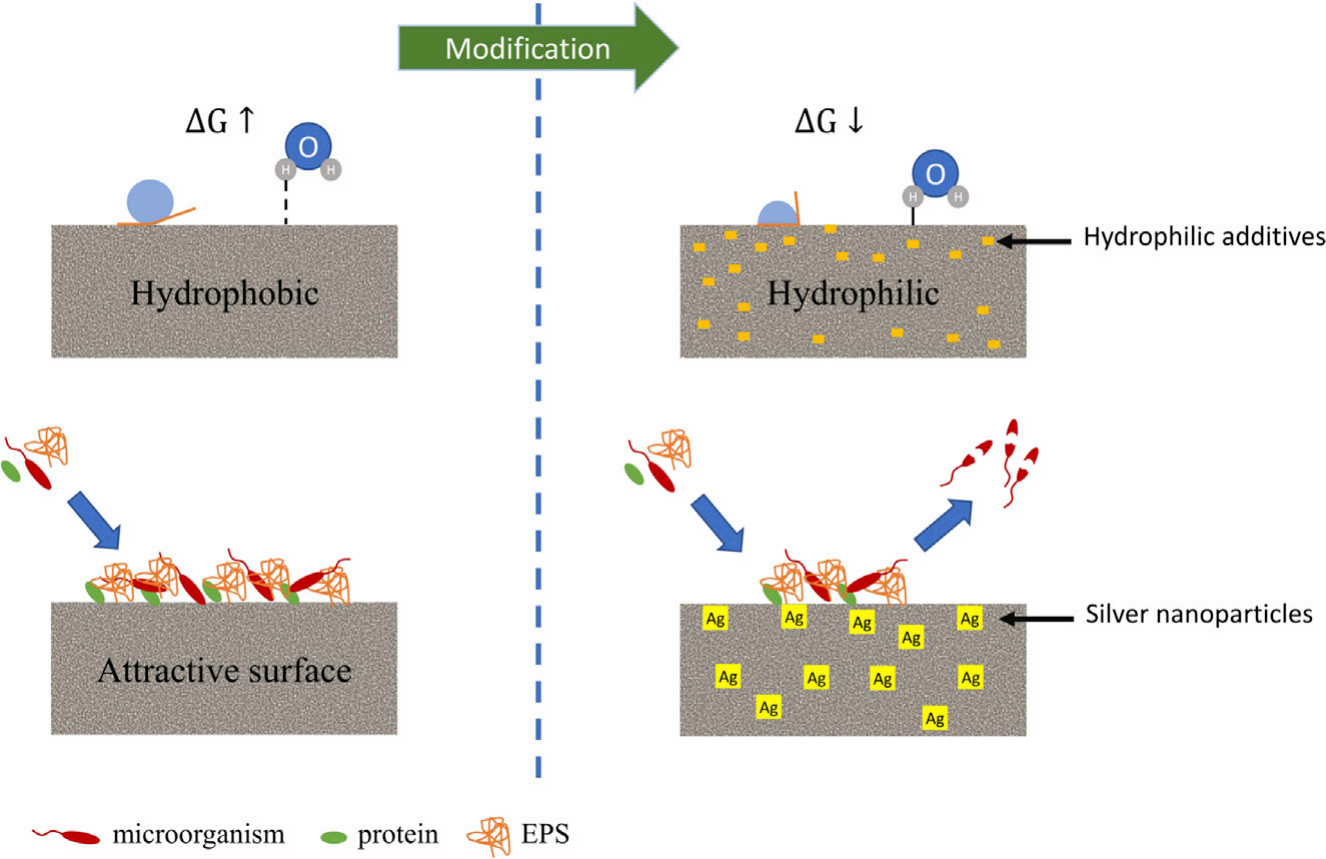
Schematic representation of antibiofouling approaches by matrix modification

The assessment of the hydrophobic-hydrophilic properties of the membranes is based on the measurement of the contact angle between the liquid-membrane interface. Membranes commonly used in MFCs are most often made of hydrophobic materials and therefore have high contact angle values (Figure 2). These materials are susceptible to adsorption of pollutants present in the medium. This is because the molecules that come from the medium will accumulate on the hydrophobic surface, minimizing the interfacial tension between the water and the membrane (Pichardo-Romero et al., 2020). It is known that membranes with hydrophilic properties are less prone to biofouling because of fewer interactions between microorganisms and the membrane surface (Elangovan and Dharmalingam, 2018),(Ghasemi et al., 2013). As shown in Figure 2, the lack of hydrogen bonds between water molecules and the surface of the membrane surface is the main cause of biofouling on the surface of a hydrophobic membrane. Repulsion of water molecules from the hydrophobic membrane surface causes an increase in entropy and, therefore, microorganisms tend to adsorb onto the membrane surface. In contrast, the hydrophilic membrane can form hydrogen bonds with water molecules, resulting in a thin water layer between the membrane surface and the electrolyte (Kochkodan and Hilal, 2015). This layer reduces the risk of the adsorption of microorganisms on the membrane surface.

There are several ways to increase the hydrophilicity of membranes used in bioelectrochemical systems. One of these strategies, shown in Figure 2, is to reduce the contact angle by doping the matrix. An experiment conducted by Kamaraj et al. (Kamaraj et al. (2015) proved that the reinforcing of the Nafion membrane with polyvinyl alcohol (PVA) nanofibers increased its hydrophilicity. The characterization of the membranes in single chamber microbial fuel cells showed that the Nafion-PVA-15 membrane provided a 15% higher maximum power density and 33% lower membrane resistance compared to commercial Nafion-117. In another study reported by Nagar et al. (Nagar et al. (2019) hydrophilicity was increased by adding hydrophilic zeolite 4A to the hydrophobic polyvinylchloride (PVC) matrix. An increase in proton conductivity values was observed with increasing concentrations of zeolite 4A in the PVC matrix until an optimal proton conductivity of 0.13 S/cm was achieved at a loading of 15% (w/w). SEM images of the membrane doped with zeolite 4A revealed low adherence of bacteria to the surface because of the high hydrophilicity of the membrane. As a result, the performance evaluation of the PVC-Zeolite membrane in the MFC showed twice the power density compared to Nafion 117. Angioni et al. (Angioni et al. (2016) obtained similar results by modifying the Nafion membrane using SBA-15 silica functionalized with SO_3_H groups as filler. The composite membrane was characterized by higher proton conductivity and a lower contact angle than Nafion 117 and a negative Zeta potential. The MFCs with a Nafion/SBA-SO_3_H10 membrane were characterized by almost three-fold higher power density than the MFCs with Nafion-117 as a membrane, after 90 days of operation.

Another group of additives that show a beneficial influence on increasing membrane hydrophilicity are carbon materials such as graphene oxide (GO) or functionalized graphene oxide (FGO). Elangovan & Dharmalingam (Elangovan and Dharmalingam, 2016) obtained a quaternized polysulfhone (QPSU) membrane modified with 1% (w/w) FGO, which demonstrated increased hydrophilic and antifouling properties compared to the commercial AMI-7001 membrane. MFCs equipped with QPSU-FGO membranes produced twice the power density of MFCs in the configuration with AMI-7001. Khilari et al. (Khilari et al. (2013) produced a composite PEM membrane using polyvinyl alcohol (PVA) and silicotungstic acid (STA) mixed with GO at concentrations ranging from 0.3% to 0.9% (w/w). With increasing GO loading, proton conductivity also increased, and the maximum proton conductivity of 0.072 S/cm at 0.5% GO loading was higher than for Nafion 117. Furthermore, three-time lower protein content was observed on the PVA-STA-GO-0.5 membrane surface compared to Nafion 117 after 76 days of operation. Similar results were also achieved in a study conducted by Li et al. (Li et al. (2019), who synthesized a new type of composite membrane by combining sulfonated graphene oxide (SGO) with poly (vinylidene fluoride) -g-poly (styrene sulfonic acid) (PVDF-g-PSSA) copolymer at various concentrations from 0.1% to 2% (w/w) SGO. At a concentration of 1% (w/w), SGO achieved higher conductivity (0.083 S/cm) and hydrophilicity (contact angle of 70.78°) than Nafion 117. The MFC configuration with SGO/PVDF-g-PSSA-1.0 exhibited the highest power density and the best power generation stability after 3 months. Ahilan et al. (Ahilan et al. (2019) also obtained the highest hydrophilic properties at a concentration of 0.5% (w/w) GO in a ceramic membrane based on polysiloxane, and when the authors examined a polysiloxane-based ceramic membrane modified with multiwall carbon nanotubes (MWCNT), an increase in the hydrophilicity of the membrane was also observed. On the other hand, the oxygen permeability between the cathode and the anode was higher than for the undoped membrane, which significantly decreased the overall performance of the modified MFCs.

**Figure 3:**
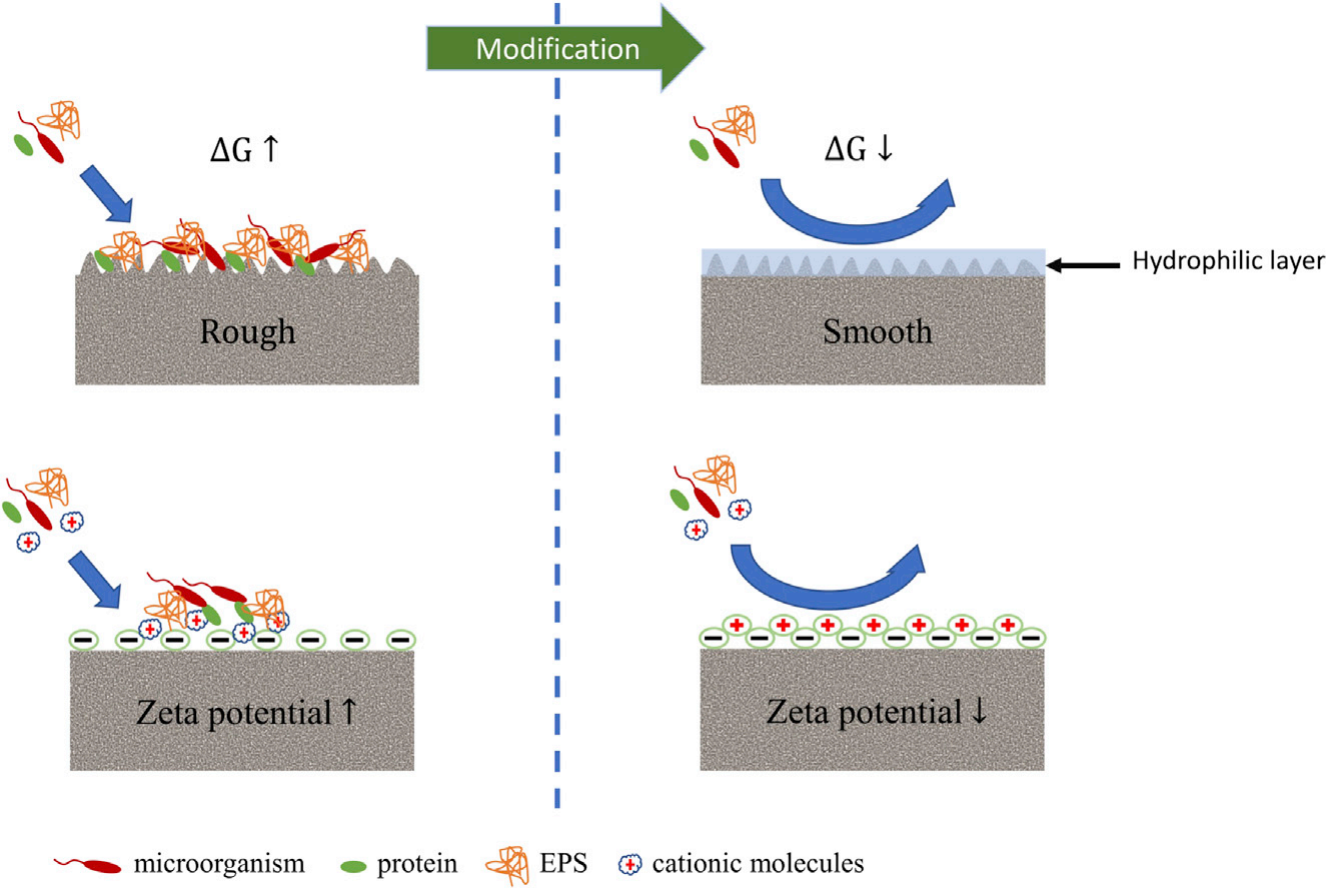
Schematic representation of methods for resisting biofouling by surface modification

Several experiments also showed a correlation between biofouling and membrane surface topology. As shown in Figure 3 greater roughness of the membrane surface increases the total surface area to which pollutants can adhere, whereas the ridge-valley structure promotes the accumulation of pollutants on the surface (Kochkodan and Hilal, 2015). Ghasemi et al. (Ghasemi et al. (2012) fabricated a composite membrane using Nafion as a matrix, doped with activated carbon nanofibers (ACNF). The decreased roughness of this membrane reduced the transfer of oxygen from the cathode to the anode and mitigated membrane fouling problems. As a result, the MFC with ACNF-Nafion achieved 1.5 times higher power density compared to Nafion 117. In another study reported by Elangovan & Dharmalingam (Elangovan and Dharmalingam, 2017b), the effect of membrane roughness on the deposition of microorganisms was investigated. They synthesized a quaternized polysulfone membrane (QPSU) membrane with a roughness of 0.29 ± 3 μm and compared its performance in an MFC with an AMI-7001 membrane with a roughness of 0.82 ± 5 μm. After 62 days of operation, they obtained a 30% higher power density in the MFC with the QPSU membrane compared to the AMI-7001. Nevertheless, these two membranes were not identical in their chemical composition, making the comparison more difficult to draw conclusions from.

Another way to improve the antifouling properties of membranes is by doping with biocides (Figure 2). Silver nanoparticles directly alter the functions of microorganisms and destabilize the plasma membrane of bacterial cells. An experiment conducted by Ben Liew et al. (Ben Liew et al., 2020) demonstrated that the addition of silver particles has a beneficial effect on reducing membrane biofouling. They fabricated a composite membrane by adding silver graphene oxide (AgGO) and graphene oxide (GO) to a sulfonated polyether ether ketone (SPEEK) matrix. The proton conductivity of the AgGO-GO-SPEEK membrane was 54.2% higher and the oxygen diffusion coefficient was 76.7% lower than for the Nafion 117 membrane. The MFC equipped with this membrane obtained the highest power density after 100 days of operation, and the results of the EIS analysis exhibited a 16% lower increase in membrane resistance compared to Nafion 117.

Frattini et al. (Frattini, Accardo and Kwon, 2020) synthesized a ceramic membrane from barium, cerium, and gadolinium oxide (BCGO) powders co-doped with 5 mol % of cobalt. Research revealed 20% better antibiofouling properties than Nafion 117. Kondaveeti et al. (Kondaveeti et al. (2018) assessed the long-term stability of inexpensive non-woven fabrics (NWF) of polypropylene membranes. After 280 days of operation, the PP80 membrane showed a lower decrease in power density compared to Nafion 117.

### The *ex-situ* methods - surface modification of existing membranes

Chemical treatment is a simple way to reduce membrane biofouling. The use of reagents that form hydrophilic groups such as -OH and -NH_2_ on the surface of a membrane increases its antibiofouling properties. For example, Ghasemi et al. (Ghasemi et al. (2013) used a three-stage treatment of the Nafion 117 membrane consisting of boiling in distilled water, then in 3% H_2_O_2_, and finally in 0.5 M H_2_SO_4_. The process improved the antibiofouling properties and increased the proton conductivity. As a result, the power density doubled compared to the MFCs equipped with pristine Nafion 117. Similar conclusions were obtained from a study conducted by Elangovan & Dharmalingam (Elangovan and Dharmalingam, 2017a), in which biological contamination of the poly(ether imide) (QPEI) membrane was counteracted by surface modification with ethanolamine (AEOH). The improved antibiofouling properties were attributed to increased hydrophilicity as a result of the incorporated -OH and -CONH groups on the surface.

A relatively easy membrane modification technique was recently proposed by Pasternak et al. (Pasternak et al. (2021). The authors recycled waste polypropylene (PP) to act as a coating on the surface of two types of ceramic membranes in MFCs. Long-term studies revealed that the PP coating improved the MFC performance by 600% compared to its non-composite counterpart. Biofouling studies were carried out with SEM / EDS, TGA, CFU, and electrochemical techniques, indicating that 373-clay possessed strong antifouling properties. The PP layer impacted the dynamic surface properties and introduced a hydrophobic layer over the porous ceramic structure.

Elangovan & Dharmalingam (Elangovan and Dharmalingam, 2018) improved the antifouling potential of the quaternized poly(ether ether ketone) (QPEEK) membrane by coating it with a polydopamine layer (PDA). Furthermore, the authors investigated the effect of polydopamine concentration on surface hydrophilicity and roughness of the surface using coatings in polydopamine solutions with concentrations ranging from 0.5 g/L to 2 g/L. The obtained data showed that the contact angle decreased rapidly with increasing polydopamine concentration, up to a concentration of 1.0 g/L and was almost the same as that modified by a 2 g/L polydopamine solution. Furthermore, profilometer tests showed the roughness was the lowest when QPEEK was coated with a polydopamine solution of 1 g/L (0.41 μm). Antifouling properties were evaluated by comparing the performance of MFCs equipped with modified membranes and AMI-7001 during operation. The lowest decrease of power density in time and the lowest protein content were recorded for the QPEEK-1.0 membrane. However, increased hydrophilicity is often not the only factor in preventing biofouling. Many studies have also shown that surface charge, as determined by measuring the Zeta potential, plays an equally important role in enhancing antibiofouling properties (Lu et al., 2020) (Yang et al., 2016). The surface of the membranes used in MFCs is often negatively charged by sulfo and carboxyl groups. A negatively charged membrane surface is less prone to bioadhesion than a positively charged surface because it repels negatively charged bacterial cells. On the other hand, as the negative charge increases, the adsorption of the cations increases, which act as bridges between the membrane surface and the bacterial cell (Figure 3) (Kochkodan and Hilal, 2015). Thus, the mixed surface charge will show the greatest ability to repel contaminants. Lu et al. (Lu et al. (2020) proposed another method to mitigate biofouling, using an FO membrane modified with silver nanoparticles (AgNP). The membrane characterization revealed increased hydrophilicity and more negative Zeta potential than that of a pristine membrane, enhancing the repulsion between the membrane and the contaminants. In addition, AgNP disturbed the functions of microorganisms. By using a combined approach, where hydrophilic polymer-silver nanoparticles composite coating is being used, it is possible to reach a synergistic effect, where the desired hydrophilicity and Zeta potential, as well as the biocidal effect on the microorganisms, can be reached. This strategy was carried out in a study conducted by Yang et al. (Yang et al. (2016), in which they reduced the surface charge of the forward osmosis (FO) membrane from −11.78 mV to −24.07 mV by applying a composite coating of silver nanoparticles (nAg) with polydopamine (pDA). At the same time, the osmotic microbial fuel cell (OsMFC) with the nAg-pDA coated membrane exhibited a 30% lower flux decline than the OsMFC with a pristine membrane because of the bactericidal properties of silver nanoparticles. In addition, the reduced contact angle of the membrane with the nAg-pDA composite layer had reduced internal resistance, resulting in a 12% improvement in power density. A dual role against the adhesion of microorganisms to the membrane surface was also indicated by Park et al. (Park et al. (2020). The combined use of PDA and AgNP showed a higher antifouling effect and long-term hydrogen recovery by a microbial electrolysis cell in comparison to a single coating. The authors also investigated the effect of the coating sequence on AgNP release into the medium. The results indicated that the coating of AgNP immediately after the PDA coating produces the lowest release of AgNP and more uniform distribution of silver nanoparticles. As a result, the PEM/PDA_Ag 0.035 membrane showed a reduction of biological contamination by 80.74% compared to the pristine PEM.

In conclusion, to enhance the antifouling properties, the membranes used in bioelectrochemical systems should have a smooth surface, more negative Zeta potential and a hydrophilic character. In addition, they may also have biocidal agents incorporated into the surface. By using one of the modification methods (Table 2), most often, more than one membrane property is being changed. Therefore, the final resistance of the separator to biological contamination will be affected by the joint effect of these properties.

**Table 2:** Comparison of different membrane modification strategies for use in MFC as a separator and their ability to control biofouling

MFC type	Membrane type	Power density, mW/m^2^	Reference membrane power density, mW/m^2^	Strategies of modification	Effect on biofouling	Lifespan	Reference
OsMFC	FO	61,5	55,2 (pristine FO membrane)	nAg-pDA coating	28% lower flux drop, 35% lower internal resistance drop after 5^th^ cycles	11 days	(Yang et al., 2016)
MEC	PEM	NA	NA	AgNP and PDA with different application sequences	80% reduction in biofouling, 60% lower drop in hydrogen production after 6 months of operation	6 months	(Park et al., 2020)
SCMFC	Ceramic membrane	NA	NA	Ceramic membrane made of barium, cerium, and gadolinium oxide powders doped with lithium or cobalt	Lower biofouling compared to Nafion 117 for cobalt doping	NA	(Frattini, Accardo and Kwon, 2020)
DCMFC	PVC	250	92 (PVC) 125 (Nafion 117)	Zeolite 4A incorporated in PVC matrix	Low bacterial attachment due to the high hydrophilic and antibacterial nature of zeolite	17 days	(Nagar et al., 2019)
OsMFC	FO	3,67 (W/m^3^)	3,45 (W/m^3^, pristine FO membrane)	AgNP modified membrane	Increased hydrophilicity, more negative zeta potential, better antibacterial property	760 h	(Lu et al., 2020)
DCMFC	PEM (Nafion 117)	100	52,8 (Nafion 117)	Boiled in distilled water, 3% hydrogen peroxide, and 0.5M of sulphuric acid	Increased hydrophilicity, two times increased COD removal	NA	(Ghasemi et al., 2013)
DCMFC	Poly(vinylidene fluoride)-g-poly(styrene sulfonic acid) copolymer (PVDF-g-PSSA)	180,27	132 (Nafion 117)	Composition of PVDF-g-PSSA with sulfonated graphene oxide (SGO)	Increased hydrophilicity, lower increase in internal resistance, and lower decrease in power density compared to Nafion 117 after 3 months of operation	3 months	(Li et al., 2019)
DCMFC	Polymer derived ceramic membrane	7,23 (W/m^3^)	6,73 (W/m^3^, Nafion 117)	Adding graphene oxide (GO) and multi-wall carbon nanotubes (MWC-NT) into a polysiloxane matrix	Increased hydrophilicity and higher coulombic efficiency of the GO-doped membrane than for Nafion 117	15 batch cycles (45 days)	(Ahilan et al., 2019)
SCMFC	Quaternized poly(ether imide) (QPEI)	620	580 (AMI-7001)	QPEI modified with ethanol Amine (4% AEOH)	Increased hydrophilicity, reduced surface roughness, decreased power, density decrease and six times lower protein content on the surface compared to AMI-7001	10 batch cycles	(Elangovan and Dharmalingam, 2017a)
SCMFC	Quaternized poly(ether ether ketone) (QPEEK)	918	578 (AMI-7001)	Coating with polydopamine (PDA)	Increased hydrophilicity, decrease in power density decrease and seven times lower protein content on the surface compared to AMI-7001	10 batch cycles	(Elangovan and Dharmalingam, 2018)
SCMFC	AEM	5,42 (W/m^3^)	3,52 (W/m^3^)	KOH-doped composite polyvinyl alcohol-polydiallyldimethylammonium chloride (PVA-PDDA)	High antimicrobial activity of quaternary ammonium moieties, more than five times lower protein on the surface compared to Ralex after 41 cycles	41 batch cycles	(Pandit et al., 2014)
DCMFC	Graphene oxide-sulfonated poly(ether ether ketone) (G0-SPEEK)	1049	1013 (Nafion 117)	Modification with silver graphene oxide (AgGO-GO-SPEEK)	16% lower increase in internal resistance and 17% lower decrease in power density than Nafion 117 after 100 days of operation	100 days	(Ben Liew et al., 2020)
DCMFC	PEM	106,7	132 (Nafion 117)	Sodium styrene sulfonate with ozone-preactivated poly(vinylidene fluoride) copolymer (PVDF-g-PSSS)	Less adherence of BSA protein than in Nafion 117	NA	(Li et al., 2017)
SCMFC	QPSU (quaternized polysulfone)	1036 ± 15	576 (AMI-7001)	Modification with functionalized graphene oxide (FGO)	Increased hydrophilicity of the membrane which eventually reduced the biofouling event in 60 days of operation	60 days	(Elangovan and Dharmalingam, 2016)
SCMFC	PEM	108	64,5 (Nafion 117)	Graphite oxide-poly(vinyl alcohol)-silicotungstic acid composite membrane (GO-PVA-STA)	Increased hydrophilicity, more than 3 times lower protein content on the membrane surface than for Nafion 117 after 76 days of operation	76 days	(Khilari et al., 2013)
SCMFC	PP	280	260 (Nafion 117)	Nonwoven fabrics of polypropylene (PP80)	Lower decrease in power density and lower carbonaceous substance content than Nafion 117 after 280 days of operation	280 days	(Kondaveeti et al., 2018)
DCMFC	Nafion	57,64	13,99 (Nafion 112) 38,30 (Nafion 117)	Activated carbon nanofiber (ACNF) and nafion nanocomposite	Less surface roughness resulted in a reduction in biofouling on the membrane	NA	(Ghasemi et al., 2012)
SCMFC	Nafion 117	75	12 (Nafion 117)	Composite membrane based on nafion 117 with SBA-15 silica functionalized with SO_3_H groups (Nafion/SBA-SO_3_H10)	Three times higher power density after 90 days of operation compared to Nafion 117	90 days	(Angioni et al., 2016)
SCMFC	Nafion	91 ± 1	79 ± 4 (Nafion 117)	Composite Nafion membrane reinforced with poly(vinyl alcohol) nanofiber (Nafion-PVA-15)	Slower decrease in power density due to fouling on membrane compared to the nafion 117	450 h	(Kamaraj et al., 2015)
SCMFC	AEM	810	575 (AMI-7001)	Quaternized polysulfone membrane synthesis (QPSU)	Reduced surface roughness of the QPSU membrane caused less biofouling formation compared to AMI-7001 after 62 days of operation	NA	(Elangovan and Dharmalingam, 2017b)
SCMFC	Ceramic membrane	81	18 (CMI-7000)	Coating with recycled polypropylene (PP80)	Higher stability of surface properties over time and higher power efficiency compared to the control ceramic membrane without modification	81 days	(Pasternak et al., 2021)

SCMFC – single chamber MFC, DCMFC – dual chamber MFC, OsMFC – osmotic MFC, NA – not available.

## Physical and chemical approach

**Figure 4:**
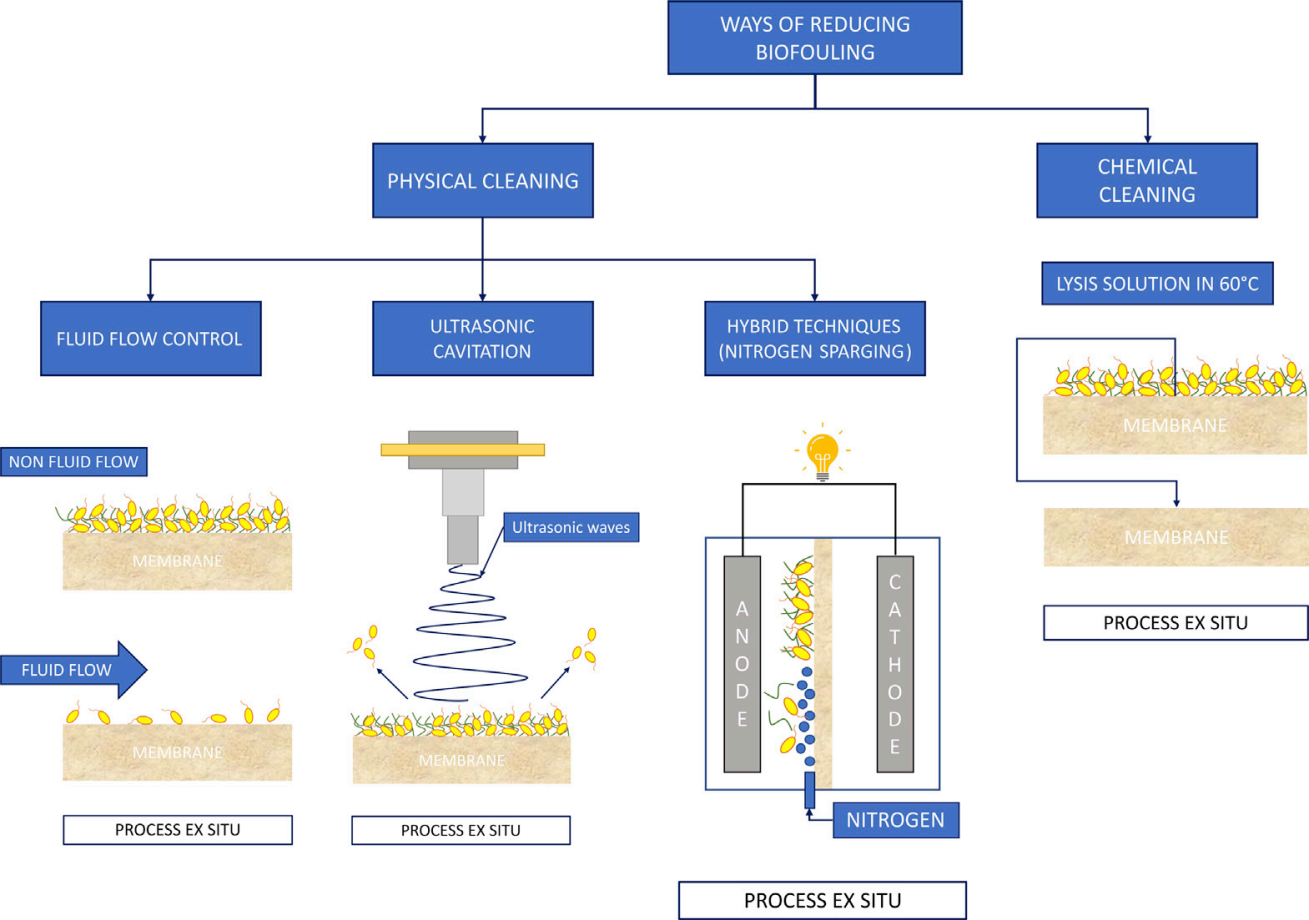
Physical and chemical approaches to inhibit and remove the biofouling layer

A commonly used approach is to reduce the effects of biofouling on the membrane surface through the application of physical or chemical agents, as well as the control of the process design (Table 3). In this chapter, we will present these strategies and discuss possible, upcoming technologies that can be applied in BESs (Figure 4).

**Table 3:** Biofilm removal strategies in bioelectrochemical systems

MFC type	Membrane type	Applied strategy	Observed effects	Reference
DCMFC	PEM (Nafion)	Membrane replacement	Increased Coulombic efficiency, from 45.1% to 59.3%	(Choi et al., 2011)
OsMFC	PEM (Nafion)	Ultrasonic waves	Restoration of the power performance (2.87 ± 0.09 Wm^-3^), corresponding to 84% of the initial value	(Xue et al., 2021)
SCMFC	IEM	UV radiation	Power density increase, from 116.2 to 198.6 mWm^-2^	(Zhang, Hui and Han, 2015)
SCMFC	IEM	Solution of 0.06 M hydrochloric acid	Power density increase, from 116.2 to 338.1 mWm^-2^	(Song et al., 2015)
DCMFC	PEM	50 mM H_2_SO_4_ solution	CE increase, from 4 to 31.5%	(Choi et al., 2011)
OsMFC	CEM	Solution of 0.1% NaOH +0.2% HCl	Flow rate increase to 73,5% of the initial value	(Xue et al., 2021)
OsMFC	CEM	0.2% NaClO solution	Regeneration of power to 3.35 ± 0.67 Wm^-3^, corresponding to 98% of the initial value	(Xue et al., 2021)
SCMFC	Ceramic	Lysis solution (0.2 M NaOH, 0.1% Triton X-100), heated to 60°C	Regeneration of power performance to 105.3 ± 16.3 μW, corresponding to the initial value of the MFCs	(Pasternak et al., 2016a; 2016b)
DCMFC	PEM	10 mM SDS solution and a 5mM NaOH solution with additional step 60 mM HCl solution to dissolve the remaining salts	Power output regenerated to its initial value	(Liu et al., 2018)

SCMFC – single chamber MFC, DCMFC – dual chamber MFC, OsMFC – osmotic MFC.

### Physical methods for biofilm reduction

Flimban et al., in their article, attempted to physically wash the proton exchange membrane. They used the Nafion 117 membrane, which was colonized by biofilm while working in a dual-chamber microbial fuel cell (TCMFC). Before washing, they recorded a decrease in cell power from 4.8 μW to 1.9 μW throughout the experiment (Flimban et al., 2020). However, physical washing of the membrane was ineffective in bringing the system back to the original power level, with only a slight increase of the Coulombic efficiency recorded after the MFC was reassembled. It has been suggested that in the case of PEM, physical cleaning should be done every six or seven months but only as a temporary and laboratory-based method. The authors suggested looking for other ways to regenerate PEM performance without removing the membrane from the MFC (Flimban et al., 2020). In contrast, Choi et al. suggested that the most effective way to regain the lost power is to replace the membrane with a new one. In the case of identical TCMFCs that were used in their studies, using the Nafion 117 membrane, the Coulomb efficiency increased from 45.1% to 59.3% after installing the new PEM (Choi et al., 2011).

Rossi et al. conducted an experiment with another approach to physical cleaning; they built a single-chamber fuel cell, where the magnet was mounted in the anode space on the inner side of the cell. An identical magnet was also introduced on the cathode side. The method consisted of physically peeling the fouling layer from the membrane by using pressure obtained from the moving magnets. After a month of testing, it was observed that the cell with regular cleaning applied to the inner side of the membrane reached power values of 116 ± 4 mW/m^2^. Similar values were observed at the beginning of the MFC operation. This is 42% better than the control cell in which the membrane was not cleaned (Rossi et al., 2018). However, fluid movement could also affect the diffusion of fuel into the biofilm matrix (Ieropoulos et al., 2015).

In another study, ultrasonic waves were used to clean the surface of the membrane from fouling and biofouling. Ultrasonic waves with frequencies above 18 kHz, propagating in an elastic medium, allowed the formation of small bubbles filled with steam. The vesicles were then enlarged and compressed alternately. When the bubble size was sufficiently large, it reached a critical value. Bubbles of the right size, when suddenly compressed, caused the phenomenon of cavitation on the surface of the membrane (Aghapour Aktij et al., 2020). Ultrasonic cleaning of CEM membranes has also been tested in an osmotic microbial fuel cell (OsMFC). Ultrasonic waves improved the values of the power generated by the MFC to 2.87 ± 0.09 Wm^-3^, which was close to the value obtained in a test with a new membrane - 3.42 ± 0.18 Wm^-3^ (Xue et al., 2021) As a result, contaminated membranes were cleaned in both experiments. Nonetheless, the lifetime of the membranes after such cleaning should be carefully assessed when using ultrasound as a long-term technique for membrane maintenance.

#### Plasma cleaning and UV radiation

If sufficient energy is provided to one or more gasses in a chamber, the breakup of atomic links is reached, with the formation of electrons, positive ions, radicals, and UV radiation. This condition is called plasma and it has been described as the fourth state of matter. Depending on the plasma-generating mechanism (e.g., plasma jet, dielectric barrier discharge, etc.), plasma systems are sources of positive and negative ions, reactive atoms, and molecules (e.g., atomic oxygen, ozone, superoxide, and oxides of nitrogen), intense electric fields, and UV radiation. In many cases, the plasma system provides a mixture of all of the physicochemical properties listed before, which can physically etch the surfaces being cleaned, removing bacteria, viruses, proteins, and any other types of organic carbonaceous material. The ionized gas reacts chemically, the UV radiation breaks the links of complex organic molecules, and the energy of the various components carries out a kind of micro sandblasting. Plasma is therefore an optimal tool for surface cleaning, surface activation, etching, and deposition of focused layers.

Plasma cleaning has the ability to effectively remove all organic contamination from surfaces through the process of a chemical reaction (O_2_ plasma) or physical ablation (argon plasma). Plasma cleaning can be applied to an array of materials, including surfaces with complex geometries. It is commonly accepted that N_2_-plasma ensures the increase of nitrogen content in carbon materials (Vaagensmith et al., 2017),(Massaglia et al., 2021), whereas O_2_-plasma is usually used for etching and surface cleaning (Boudou et al., 2003).

Several works in the literature ((Sun and Bijweb, 1989),(Tiwari et al., 2011)) have shown that O_2_/N_2_ air-plasma induces two concurrent effects: I) removal of surface atoms or clusters of atoms induced by etching reactions driven by O_2_ and II) further reactions between reactive sites and the reactant N_2_ species in the plasma, forming N-doping active sites. Plasma cleaning improves material biocompatibility or bioactivity and removes contaminating proteins and microbes from surfaces. It can act as a chemical-free means of adding biologically relevant functional groups (carbonyl, carboxyl, hydroxyl, and amine) to material surfaces (Lerman et al., 2018). Plasma cleaners are a general tool in life sciences, being used to activate surfaces for cell culture (Pratt, Williams and Jarrell, 1989) and tissue engineering (Beardslee et al., 2016).

Contaminants on the surface of the material to be treated are turned into vapor, so no residues are left on the surface, leaving the latter in an ultrafine clean state. The plasma cleaning process works under atmospheric pressure. Its advantages compared to standard chemical and vacuum plasma cleaning processes include ultrafine cleaning, no residues, no wet chemistry, gentle surface treatment, air or nontoxic working gases, no expensive vacuum equipment, and fast cleaning giving promotion of wetting and adhesion if required. A search of the literature shows that although plasma treatments have been used to achieve highly active catalysts for the oxygen reduction reaction (ORR) in MFC by treating cathodes (Massaglia et al., 2021), their use for cleaning membranes has not yet been reported. However, this section on plasma cleaning is included in this review because of its potential advantages over all other methods of cleaning and the high likelihood that it will be a successful method if used in the future.

Another way to clean the membrane and prevent the negative effects of biofouling is to use UV radiation to clean the surface of the membrane. Song et al. demonstrated in their article that the irradiation of an ion exchange membrane with ultraviolet waves can positively affect its efficiency. The power of single-chamber fuel cells that were irradiated with UV rays increased from 116.2 mWm^-2^ to a value of about 198.6 mWm^-2^(Zhang, Hui and Han, 2015). The authors also pointed out that this was not one of the most effective ways to regenerate the membrane.

### Chemical methods

A common approach is to use chemicals for biofouling removal and prevention of biofouling. One of the most popular cleaning agents for membrane surfaces is hydrochloric acid. Song et al. (Song et al. (2015) showed that after immersing the membrane in 0.06 M hydrochloric acid solution, followed by rinsing with distilled water, the MFC efficiency was brought back close to the initial values. The power output of the single-chamber MFC before regeneration was 116.2 mWm^-2^, whereas after regeneration, it reached 338.1 mWm^-2^.

The influence of biofouling on Coulombic efficiency was investigated using TCMFCs with CEM membranes, where 50 mM H_2_SO_4_ acid was used to chemically remove biofouling from a Nafion surface. After the treatment, CE increased from 4 to 31.5% which was comparable to the new membrane (Choi et al., 2011). In another study, chemical cleaning of the CEM membrane was carried out by using two types of these solutions: 0.1% NaOH +0.2% HCl and 0.2% NaClO. In both cases, an improvement in the power density generated by the OsMFC was observed. The highest value of the power density was recovered by a 0.2% NaClO solution, being 3.35 ± 0.67 Wm^-3^, whereas 3.42 ± 0.18 Wm^-3^ was recorded for a new membrane. Furthermore, in the case of 0.2% NaClO solution, the flow rate of distilled water through the MFC reached 91% of the original value. In the case of 0.1% NaOH +0.2% HCl, the resulting flow rate was 73.5% of the initial flow rate (Xue et al., 2021).

In other research, Pasternak et al. (Pasternak et al., 2016a; 2016b) also showed a positive effect on the use of chemical agents against biofouling. For this purpose, a two-stage membrane/cathode assembly regeneration method was used. The first stage of regeneration consisted of washing the cathode with a lysis solution (0.2 M NaOH, 0.1% Triton X-100), heated to 60°C. The second stage of regeneration consisted of removing the outer cathode layer and reapplying the material with an identical carbon loading. In the whole process, power densities of 105.3 ± 16.3 μW were obtained, which corresponded to the initial value of the MFC (Liu et al., 2018). Chemical cleaning of the membranes was found to be effective, although it should be noted that hydrogen peroxide and sulfuric acid are highly aggressive substances. The combination of these chemicals can be used for Nafion membranes, but other polymer membranes can be degraded and destroyed.

### Optimization of fluid flow dynamics

In recent years, the technology of computer-aided design has been greatly developed. This technology is used in many sectors such as aviation, automotive, and chemical industries and recently also to create new microbial fuel cell designs. Comsol and Ansys Fluent are the best-known programs for fluid flow modeling, and both are used in MFC research. Computational fluid dynamics (CFD) can be used not only to minimize the occurrence of dead spaces in the BESs, which translates into better coverage of the anode substrate, but also to create shear forces in the cell, which will slow down the process of biofouling on the membrane.

One of the most important criteria in the design of microbial fuel cells is that bioelectrochemical reactions take place between the solution containing the substrate and the bacteria on the anode surface. If there are dead spaces in the BESs, this may cause a local decrease in the substrate concentration and cause local biofilm dieback at the anode. Kim et al. designed 18 different microbial fuel cells that were then tested in flow modeling software (Kim et al., 2014). Flow dynamics and dead space optimization had an effect on increasing the power performance and hypothetically on reducing the rate of impurities forming on the membrane surface.

It is worth noting that numerical fluid mechanics and computer-aided design not only shorten the time of creating new projects but also optimize the costs associated with the implementation of the appropriate design. Juan D. López-Hincapie et al., in their work, designed a microbial fuel cell that was intended to act as a biosensor, using CFD to prepare the MFC model. The authors managed to speed up the COD detection method (45 ± 6.4 min vs. 63 ± 10 min) and reduce the difference in quantification compared to the traditional COD quantification method (López-Hincapié et al., 2020). CFD in MFC research is relatively underexplored and so far used primarily in the design of MFC-based small, mL-scale biosensors. This means that there is still scope for research into this technique when designing larger power cells and introducing the emphasis on biofouling prevention. The difficulties associated with modeling the phenomena occurring inside the MFC require the use of many equations and enormous computing power. However, it is a technique that can significantly improve the performance of microbial fuel cells and mitigate the effects of biofouling on the surface of membranes in the future.

## Hybrid techniques to prevent biofouling in BESs

There are many different technological processes in which membranes can be biofouled. Therefore, it is worth paying attention to hybrid solutions aimed at preventing or minimizing the effects of this phenomenon. Although some of the methods have not been used yet, for example, bioelectrochemical systems, they could become a part of future solutions.

Membrane bioreactors (MBR) or electro-membrane bioreactors (eMBR) combine biodegradation, electrochemistry, and membrane filtration into one system. This technique is providing higher wastewater quality compared to conventional activated sludge plants. One of the most serious threats to this type of device is the process of rapid biofouling on the membrane. It turns out that the use of an electric field on the surface of the membrane reduces the formation of a biofilm on its surface. As a result, the operating costs of the system are reduced (Ensano et al., 2016). However, the application of an electric field requires energy, which can be produced by MFCs and thus may become an alternative way of spending this energy for the internal needs of bioelectrochemical reactors.

In fact, a similar approach was proposed by Xu et al. (Xu et al. (2020), who integrated BES into an ultrafiltration system (UF). The UF process is highly susceptible to biofouling on the membrane surface. Integrating the conductive support over the UF membrane and poising its potential to 1.0 V led to superior filtration performance compared to the control. This was expressed by the lower membrane transpressure, EPS content, and microbial viability. The lower EPS content is a common feature for well-performing electroactive biofilm, although in typical MFC, EPS is usually substituted with a higher density of bacterial cells (Pasternak et al., 2018).

Another technique involves cleaning the membrane by aerating it with air bubbles (Ghernaout, 2020) and was successfully used in MBRs. However, when implementing it in bioelectrochemical systems, it must be considered that the bacteria in the anode chamber require anaerobic conditions. Thus, to avoid aeration of the anolyte in the anode chamber, it would be worth using a nitrogen gas stream, which would not only clean the membrane of impurities but also reduce the concentration of dissolved oxygen in the anolyte.

In the case of reverse osmosis processes, one way to limit the growth of biofilm on the membrane is to limit nutrient concentrations (Nguyen, Roddick and Fan, 2012). When the concentration of the substrates is reduced, while the flow rate is increased, it is possible to exert an additional shear force which would help slow down the process of biofilm formation on the membrane. However, it could also reduce the power output, presuming that it exceeds the specific kinetic rate of bioelectrochemical reactions.

## Conclusion

This review outlines several strategies and monitoring approaches oriented towards reducing bioadhesion to the separators used in bioelectrochemical systems. The research was based on doping with carbon materials, coating the surface with polymers, doping with silver nanoparticles, and the synthesis of new membranes with high antifouling potential. The result of these tests was higher resistance to biofouling compared to separators commonly used in bioelectrochemical systems.

In addition, a group of physical and chemical methods was identified to regenerate membranes already affected by biofouling. Among them, the chemical cleaning methods proved to be effective and easy to conduct; however, on the other hand, the chemicals used in the process are rather aggressive, may affect the other types of materials in the system, and have to be handled with care.

Depending on the type of separator, various strategies can be applied and should take into account not only the efficiency of the method but also its cost-efficiency (Table 4). In general, ceramic membranes are very resistant to aggressive chemical cleaning agents, and some low-cost surface coatings have already been proposed. The most popular ion exchange membranes are also known to be resistant to chemical attack, which makes them easy to clean, whereas their overall high cost also justifies more expensive surface treatments such as nanoparticle modifications. Synthetic polymers were also investigated and seem to be suitable for BES applications. However, their chemical resistance varies, and thus the cleaning agents have to be selected accordingly, whereas their low cost also justifies the replacement, if the BES design allows for it. The most difficult and varied group is composed of natural polymers because of their diverse properties. The safe strategy could be a dedicated surface treatment or carefully selected cleaning agents, whereas Ultrasounds could also be a good alternative for those polymers which present relatively strong mechanical properties.

**Table 4:** Guidelines for choosing the most commonly used membranes in BES and combating biofouling phenomenon

Separator type	Membrane examples and their cost, EUR/m2	Example membrane durability prior biofouling	Suggested strategy	References
Ceramic	Earthenware, 4 Mullite, 16,50 Alumina, 211	81 days for PP80 modified ceramics	Low cost surface modification (such as PP coating) and wide variety of chemical cleaning agents, including NaOH, HCl and surfactants, such as SDS and Triton X-100	(Pasternak et al., 2016a; 2016b) (Pasternak et al., 2021)
Ion exchange membranes	Zirfon, 51 CMI-7000, 340 Nafion 117, 2130	90 days for silica modified Nafion 117	Because of high cost of the membranes, surface modification with gold and silver nanoparticles is justified, chemical cleaning allowed for chemical attack resistant membranes	(Hernández-Flores et al., 2019) (Angioni et al., 2016)
Synthetic polymers	Polypropylene, 0,25 Polystyrene, 0,3	280 days for non-woven fabric polypropylene	Low cost surface modification allowed, chemical cleaning adjusted to the type of the polymer, replacement of the separator is economically justified	(Mathuriya and Pant, 2019) (Kondaveeti et al., 2018)
Natural polymers	Mixed cellulose ester, 57 Silk fibroin, 48	60 days for mixed cellulose ester filter	Dedicated surface modification, ultrasonic or chemical cleaning	(Wang and Lim, 2017)

The area of biofouling in BESs is heavily underexplored and techniques derived from other processes may be very useful in combating this problem. Such approaches may involve novel techniques of plasma cleaning, application of electric field, or utilization of computational fluid dynamics to induce shear forces. These techniques have never been tested in bioelectrochemical systems but have proved their applicability to other types of processes. Thus, the topic will certainly benefit from being extended with methods borrowed from other areas of membrane science.
